# Clinicopathological Analysis and Survival Outcomes of Radiation‐Induced Oral Squamous Cell Carcinoma: A Systematic Review and Meta‐Analysis

**DOI:** 10.1111/jop.70106

**Published:** 2025-12-30

**Authors:** Anne Evelyn Oliveira Moura, Carla Isabelly Rodrigues‐Fernandes, Ana Gabriela Costa Normando, Danielle Machado Farias, Márcio Ajudarte Lopes, Pablo Agustin Vargas, Alan Roger Santos‐Silva, Fábio de Abreu Alves, Luiz Paulo Kowalski, Danyel Elias da Cruz Perez

**Affiliations:** ^1^ Department of Oral Diagnosis, Semiology and Pathology Areas Piracicaba Dental School, University of Campinas São Paulo Brazil; ^2^ Oral Pathology Unit, Department of Clinic and Preventive Dentistry Universidade Federal de Pernambuco Recife Pernambuco Brazil; ^3^ Department of Stomatology School of Dentistry, University of São Paulo São Paulo Brazil; ^4^ Department of Stomatology A.C. Camargo Cancer Center São Paulo Brazil; ^5^ Head and Neck Surgery Department Medical School, Universidade de São Paulo São Paulo São Paulo Brazil; ^6^ Head and Neck Surgery and Otorhinolaryngology Department A.C. Camargo Cancer Center São Paulo São Paulo Brazil

**Keywords:** head and neck cancer, oral cancer, radiation‐induced cancer, radiotherapy, squamous cell carcinoma

## Abstract

**Background:**

Radiotherapy‐induced malignancies are well‐documented, but the clinicopathological profile and prognostic factors of radiation‐induced oral squamous cell carcinoma (R‐OSCC) remain unclear.

**Methods:**

The present review was conducted in accordance with the PRISMA guidelines and has been registered in the PROSPERO (registration number CRD42024560015). A comprehensive search was performed across electronic databases and gray literature sources. The risk of bias was assessed using the JBI tool. Meta‐analysis was performed with Review Manager 5.4.

**Results:**

Five cohort studies were included (*n* = 310), with four pooled in a meta‐analysis. R‐OSCC was significantly associated with tongue tumors (*p* = 0.007) and early clinical stages (*p* < 0.0001). Negative perineural invasion was linked to sporadic OSCC (*p* = 0.0008). Smoking (*p* = 0.0008) and radiotherapy (RT) history (*p* < 0.00001) were associated with decreased overall survival (OS), while RT history also reduced disease‐specific survival (DSS) in R‐OSCC patients (*p* = 0.006).

**Conclusions:**

R‐OSCC predominantly affects the tongue, is diagnosed in early stages, and the history of RT is associated with reduced OS and DSS.

## 1 Introduction

Radiotherapy (RT) is among the most commonly used cancer treatments [1]. In head and neck cancer (HNC), it is used for early‐stage tumors and combined with surgery and chemotherapy in advanced cases [2]. The carcinogenic potential of ionizing radiation is well established, as it causes DNA breaks, chromosomal aberrations, mutations, and genetic instability [1, 3].

Radiation‐induced malignancies have been reported in various primary tumors [1]. RT for prostate cancer is associated with a 6% increased risk of second solid tumors [4]. In HNC, an analysis of 72 malignant parotid tumors found six cases within prior irradiation fields, occurring 2 to 24 years post‐RT [5]. Additionally, a study of 1358 patients reported a 1.8% incidence (25 cases) of RT‐induced HNC [6].

A systematic review identified 122 cases of radiation‐induced sarcomas in the oral cavity [7]. Although some case reports describe radiation‐induced oral squamous cell carcinoma (R‐OSCC), robust clinical and survival outcome data remain limited [8, 9, 10]. This study aims to synthesize existing evidence on the clinicopathological profile and prognostic factors of R‐OSCC.

## 2 Methods

### 2.1 Review Protocol

The protocol for this review was registered in PROSPERO (International Prospective Register of Systematic Reviews) under the registration number CRD42024560015.

### 2.2 Eligibility Criteria

The acronym PECOS (Population, Exposure, Comparison, Outcomes, Studies), defined as (P) Patients diagnosed with oral squamous cell carcinoma (OSCC); (E) Previous radiotherapy for head and neck cancer; (C) No history of prior radiotherapy for head and neck cancer; (O) Clinicopathological features, treatment modalities, and outcomes of patients with radiation‐induced OSCC; (S) Observational studies (cross‐sectional and cohort designs), was developed to guide the focused research question: “What are the clinicopathological profile and prognostic factors of radiation‐induced oral squamous cell carcinoma, and how do they differ from those of sporadic oral squamous cell carcinoma?”

Inclusion criteria comprised observational studies (cross‐sectional and cohort studies) that evaluated the clinicopathological of R‐OSCC. Selected articles should have considered the following diagnostic criteria [11, 12, 13]:

1. A medical history of RT in the head and neck region for other neoplasms than oral squamous cell carcinoma (OSCC).
2. The development of a new cancer of the oral cavity after RT.
3. An interval of more than 6 months between the end of radiation therapy for the primary tumor and the diagnosis of R‐OSCC.
4. The microscopic confirmation of OSCC.

Studies in English, Portuguese, and Spanish were screened. Exclusion criteria included reviews, protocols, short communications, opinions, letters, book chapters, abstracts, in vitro studies, and manuscripts without full text. Studies lacking R‐OSCC clinicopathological data or including SCC outside the oral cavity and those without individual patient data were excluded.

Case series and case reports were excluded, consistent with the initial plan outlined in the PROSPERO protocol. Given the large volume of articles retrieved, the team opted to exclude these study designs as case reports are typically recommended for rare or delayed adverse outcomes and are more prone to bias.

### 2.3 Information Sources and Search Strategy

Individualized search strategies following the PECOS strategy were performed in July 2024 and updated on June 07, 2025, for each of the following databases: Pubmed, EMBASE, LILACS, Scopus, and Web of Science. Gray literature was searched on Google Scholar (Table S1). References of included studies were manually screened for additional articles. Retrieved studies were uploaded to Endnote Web (Endnote Web, Clarivate Analytics, Philadelphia, PA), where duplicates were automatically removed. ProQuest was not used as initially planned in the PROSPERO protocol, as searches conducted in Google Scholar (gray literature) yielded a diversity of studies comparable to those anticipated from ProQuest.

### 2.4 Selection Process

Study selection occurred in two phases, independently by two reviewers (AEOM and DMF). First, titles and abstracts were screened using Rayyan [14]. Studies meeting inclusion criteria proceeded to full‐text assessment. Disagreements were resolved by discussion between reviewers, with a third author (CIRF) consulted if needed to ensure appropriate selection.

### 2.5 Data Collection and Extraction

Clinicopathological data from included publications were independently extracted by one author (AEOM) and verified by a second (DMF). A specific extraction form in Microsoft Excel was used to process qualitative and quantitative data. Extracted information included, when available: publication details (year, country, continent, study type/design); patient characteristics with R‐OSCC (sample size, sex, age, tobacco/alcohol use, tumor location, clinical stage, margin status, grade, and treatment); clinical outcomes (overall survival, follow‐up, and recurrence/metastasis). Additionally, data on the primary tumor (location, microscopic diagnosis), RT details (dose, latency, and type), sample and outcomes of sporadic squamous cell carcinoma (S‐OSCC) patients, statistical data, and main study conclusions were collected when available.

### 2.6 Risk of Bias Assessment

The risk of bias in individual studies was independently assessed by two authors (AEOM and DMF) using JBI Critical Appraisal Tools according to each study's design [15]. Studies scoring ≤ 49% “yes” were high risk, 50%–69% moderate risk, and ≥ 70% low risk. Disagreements were resolved by consensus.

### 2.7 Effect Measures and Synthesis Methods

Qualitative synthesis grouped and compared data on outcomes using Microsoft Excel. Given the limitations of the data available in the included studies, it was not possible to determine the prevalence of R‐OSCC as originally specified in the PROSPERO protocol. However, the studies provided information on both sporadic and radiation‐induced OSCC, allowing a meta‐analysis comparing these datasets.

The primary quantitative outcome was the association between clinicopathological features and R‐OSCC or S‐OSCC diagnosis. Odds ratios (OR) with 95% CI were calculated from dichotomous data of patients with features and total patients in each group. Mantel–Haenszel method with a random effects model was used for ORs.

The secondary outcome was prognostic factors of R‐OSCC, assessed by overall survival (OS) and disease‐specific survival (DSS) hazard ratios (HR) with 95% CI. Log HR and standard errors were calculated via inverse variance method in a random effects model to pool HRs.

Meta‐analyses required at least two studies with suitable data. Heterogeneity was assessed by I^2^, with > 50% considered substantial [16]. Publication bias and meta‐regression were not performed due to fewer than 10 studies [16]. Data analysis used Review Manager (RevMan 5.4, Cochrane Collaboration, 2020).

## 3 Results

### 3.1 Study Selection

**FIGURE 1:**
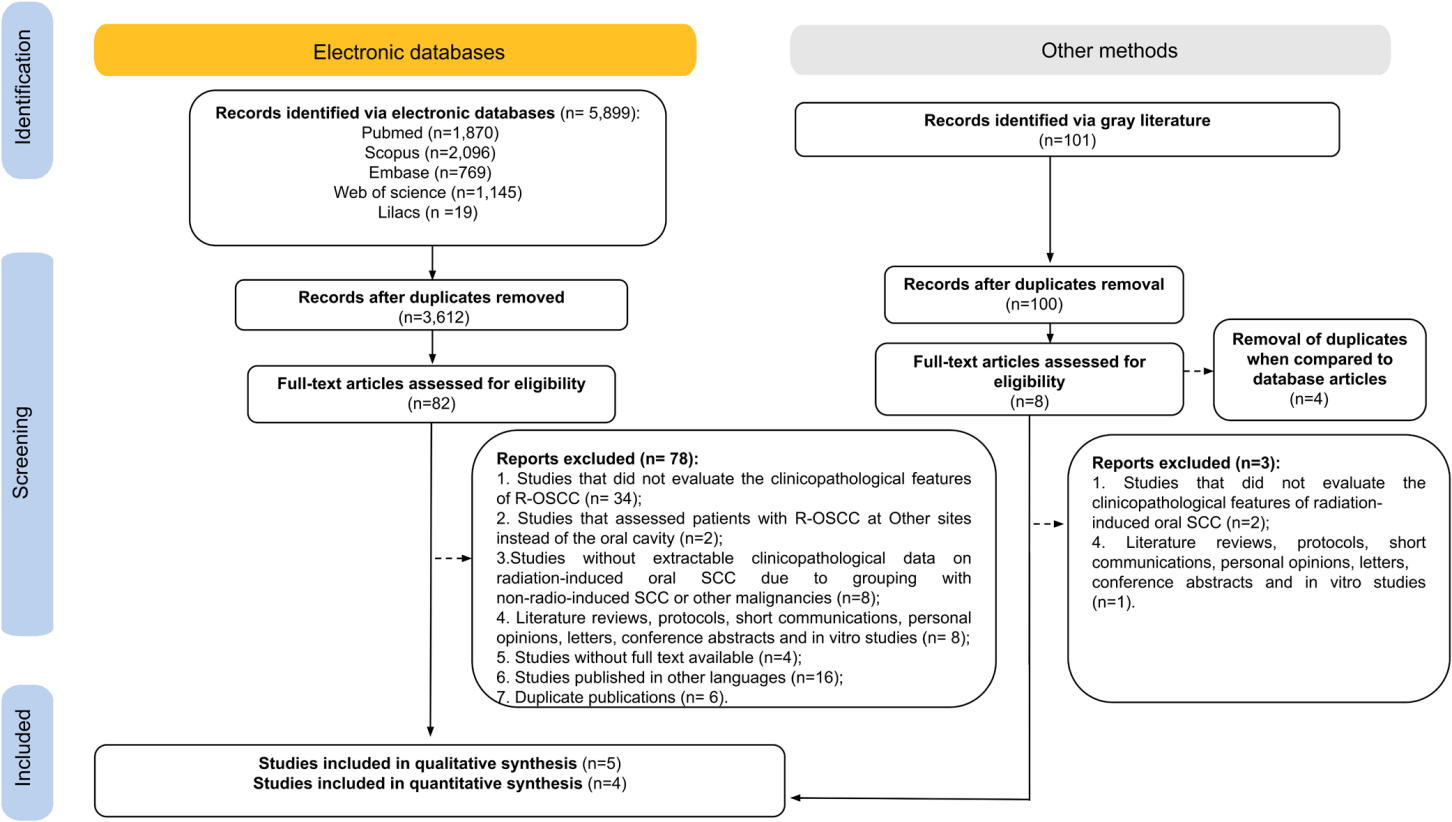
Flow diagram of the literature search and selection criteria according to PRISMA.

The searches identified 5899 database records and 100 from gray literature, totaling 5999 studies managed with duplicates removed. After this, 3711 references remained—3612 from databases and 99 from gray literature—and their titles and abstracts were screened. Following eligibility confirmation and resolving disagreements, 90 studies were selected for full‐text review. Seven studies showed potential sample overlap; Hu et al. [17] was chosen for having the largest sample, and the others were excluded to avoid duplication and ensure independence [13, 18, 19, 20, 21, 22]. Finally, five studies met eligibility for qualitative synthesis, with four included in the quantitative analysis (Figure 1). Exclusion reasons are detailed in Table S2.

### 3.2 Study Characteristics

#### 3.2.1 Publication Information and Sample Information

The included studies, all retrospective cohort designs, were published in English between 2009 [23] and 2023 [24]. They originated from China (*n* = 4) and Japan (*n* = 1). The review sample included 1697 patients: 310 (18.3%) with R‐OSCC and 1387 (81.7%) with S‐OSCC.

#### 3.2.2 Radiation‐Induced Oral Squamous Cell Carcinoma Information

A total of 310 patients were diagnosed with R‐OSCC, with study sizes ranging from 3 [23] to 116 [17]. Most were male (236; 76.1%) with a mean age of 57.2 years. Smoking data from four studies (*n* = 302) showed 27.2% of current smokers. Chow et al. [24] lacked smoking data for 5 patients, and Toda et al. [23] reported it as grouped data. Alcohol use was reported in most studies (*n* = 250), with 10% current drinkers; Chow et al. [24] did not provide this information.

All tumors were microscopically diagnosed as squamous cell carcinoma (SCC) located in a previously irradiated field for at least 6 months. The mean latency between RT and tumor was 142.1 months, ranging from 120 [17, 24] to 202.4 months [23]. One study only specified latency > 6 months as an inclusion criterion [25].

The tongue was the most affected site (194 cases; 62.6%), followed by the gingiva (49; 15.8%) (Figure S1). Treatment approaches varied. Surgery alone was most common (104 cases; 33.5%), followed by combined therapies, mainly surgery‐based. Surgery with RT was used in 32 cases (10.3%), and surgery with RT plus chemotherapy in 38 (12.3%). In 89 patients (28.7%), treatment was described as curative—surgery or surgery with adjuvant chemoradiotherapy—though only 5 (5.6%) actually received RT [17].

Most cases (178; 57.4%) were AJCC stage I or II. Neck lymph node metastasis occurred in 68 patients (22.1%), with one study missing this data [23]. Surgical margin status was available for 182 patients (58.5%), mostly negative (169; 92.9%). Most studies reported tumor differentiation grade (260 cases; 83.6%), with well‐differentiated OSCC in 126 cases (41.6%). Two studies did not report data for 7 patients [23, 24]. Song et al. [25] grouped well‐ and moderately differentiated OSCC (43 tumors; 14.2%). Perineural invasion data were available for 135 cases [24, 26], with absence reported in 63.0%. Lymphovascular invasion was identified in 100 patients [24, 26].

The mean follow‐up was 62.3 months, ranging from 25 [17] to 122.4 months [24]. Table 1 summarizes clinicopathological features; individual R‐OSCC data are in Table S3.

**TABLE 1:** Summary of clinical‐pathological characteristics of the sample of this SR with R‐OSCC.

Sample characteristic (*n* = 310)	*N*	%
Sex (*n* = 310)		
Male	236	76.1%
Female	74	23.9%
Alcohol status (*n* = 250)		
Alcoholic drinker	25	10.0%
Non‐alcoholic drinker	225	90.0%
Tobacco status (*n* = 302)		
Smoker	82	27.2%
Non‐smoker	220	72.8%
Anatomic site (*n* = 310)		
Tongue	194	62.6%
Gingiva	49	15.8%
Buccal mucosa	34	11.0%
Floor of mouth	11	3.5%
Palate	20	6.5%
Alveolus	1	0.3%
Mandibular bone	1	0.3%
Clinical Stage (AJCC) (*n* = 310)		
Stage I–II	178	57.4%
Stage III–IV	132	42.6%
Lymph node status (*n* = 307)		
N0	239	77.9%
N+	68	22.1%
Margin status (*n* = 182)		
Positive	13	7.1%
Negative	169	92.9%
Tumor microscopic differentiation (*n* = 303)		
Well	126	41.6%
Moderate	92	30.4%
Poor	42	13.9%
Grouped data	43	14.2%
Perineural invasion status (*n* = 135)		
Positive	50	37.0%
Negative	85	63.0%
Lymphovascular invasion status (*n* = 100)		
Positive	29	29.0%
Negative	71	71.0%
Treatment (*n* = 310)		
Surgery alone	104	33.5%
Surgery combined with radiotherapy	32	10.3%
Surgery combined with chemotherapy	10	3.2%
Surgery combined with radiotherapy and chemotherapy	38	12.3%
Radiotherapy alone	6	1.9%
Chemotherapy alone	23	7.4%
Brachytherapy	1	0.3%
Radiotherapy or chemoradiotherapy	2	0.6%
Curative treatment*	89	28.7%
Supportive care**	5	1.6%

Abbreviation: AJCC, american joint committee on cancer.

* Surgery or surgery plus concurrent chemoradiotherapy.

** The authors did not provide a clear definition of supportive care.

#### 3.2.3 Primary Tumor Information

Most tumors affected the nasopharynx (269 cases; 86.5%), followed by the oral cavity (27 cases; 8.7%) and oropharynx (3 cases; 1%). One study did not specify the affected head and neck region [23]. Tumors were predominantly undifferentiated nasopharyngeal carcinoma.

The mean radiation dose for primary tumor treatment was 62.2 Gy, ranging from 48.3 [23] to 70 Gy [17]. Song et al. (2021) [25] did not report this data (48 cases). RT type (conventional, 3D, or modulated) was unreported for most cases (164; 57.3%). When available, conventional RT was the most used (126 cases; 86.3%).

#### 3.2.4 Sporadic Squamous Cell Carcinoma Information

The S‐OSCC sample included 1387 patients and served as the comparison group for R‐OSCC in three studies [17, 24, 26]. Most patients were male (1063; 76.6%), with a mean age of 60.2 years. Alcohol consumption was reported in 209 cases (16.4%) and smoking in 413 (29.8%). The tongue was the most commonly affected site (659 cases; 47.5%). Approximately half of the patients were diagnosed at stages I or II (759; 54.7%). Cervical lymph node metastases were present in 445 cases (32.1%). A total of 637 patients (48.2%) had well‐differentiated squamous cell carcinoma. Perineural and lymphovascular invasion were observed in 156 (13.6%) and 166 (13.7%) cases, respectively. Combined treatment with surgery and either chemotherapy or RT was the most frequent therapeutic approach (981 cases; 70.7%). The mean follow‐up duration was 79.3 months, from 45.5 to 122.4 months (Table S4).

### 3.3 Risk of Study Bias

**FIGURE 2:**
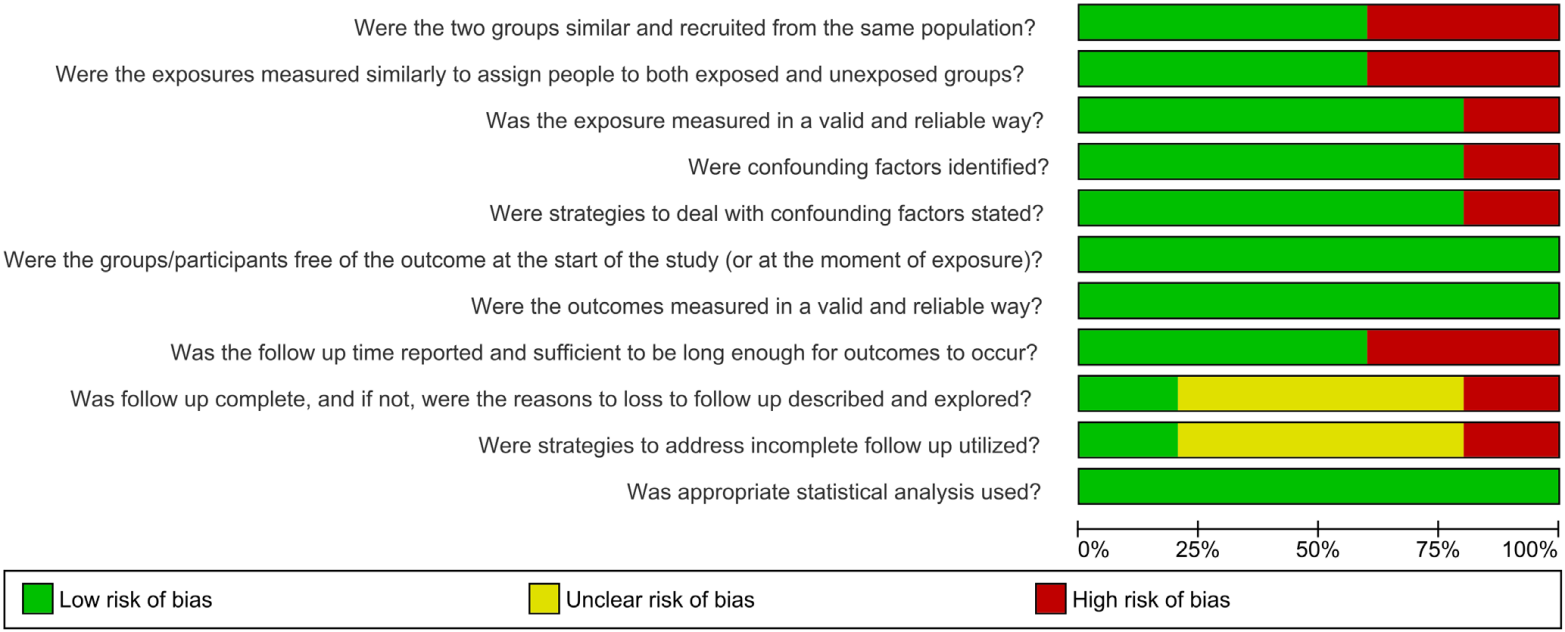
Risk of bias assessed by the JBI Critical Appraisal Tools. The risk of bias was categorized as high when the study achieved up to 49% “yes” scores, moderate when the study achieved 50% to 69% “yes” scores, and low when the study achieved more than 70% “yes” scores.

Most studies (4; 80.0%) were classified as having a low risk of bias, while one study (20.0%) was considered to have a high risk of bias [23]. The main methodological limitations identified were insufficient information regarding follow‐up and the lack of strategies to address incomplete follow‐up. Conversely, in most studies, exposure was measured accurately, confounding factors were identified, and appropriate strategies to manage them were reported (4 studies; 80%). All included studies ensured that participants were free of the outcome at baseline, measured outcomes using valid and reliable methods, and performed appropriate statistical analyses. Risk of bias is shown in Figure 2 and detailed in Table S5.

### 3.4 Results of Individual Studies

Two types of studies were included: those that compared R‐OSCC and S‐OSCC [17, 24, 26], and those that specifically described the clinicopathological and follow‐up characteristics of R‐OSCC [23, 25].

Chow et al. [24] reported that R‐OSCC patients had earlier recurrences and significantly lower recurrence‐free survival compared to S‐OSCC (*p* = 0.04). Most recurrences in R‐OSCC were locoregional, while 35.2% of S‐OSCC cases developed distant metastases. Similarly, Hu et al. [17] found worse local recurrence‐free survival in R‐OSCC (*p* < 0.05), with no significant difference in regional metastasis‐free survival (*p* = 0.112). Both studies identified prior RT as an independent predictor of poorer OS [17, 24], and Hu et al. [17] also found it to be an independent risk factor for DSS.

Dai et al. [26] reported disease‐related mortality in 72.0% of R‐OSCC cases and 75.7% of S‐OSCC cases. In the R‐OSCC group, smoking, clinical stage, and tumor differentiation were independent predictors of poor OS. For DSS, clinical stage, perineural invasion, and treatment were adverse prognostic factors.

Song et al. [25] evaluated OS and prognostic factors in R‐OSCC patients. The 3‐ and 5‐year OS rates were 60.3% and 39.4%, respectively. Margin status and extranodal extension were significantly associated with OS, but only margin status remained an independent predictor (HR = 3.976, 95% CI 1.596–9.904, *p* = 0.003). In a small series, Toda et al. (2009) [23] reported one death from R‐OSCC, with two patients alive at the end of follow‐up.

### 3.5 Results of Syntheses

**FIGURE 3:**
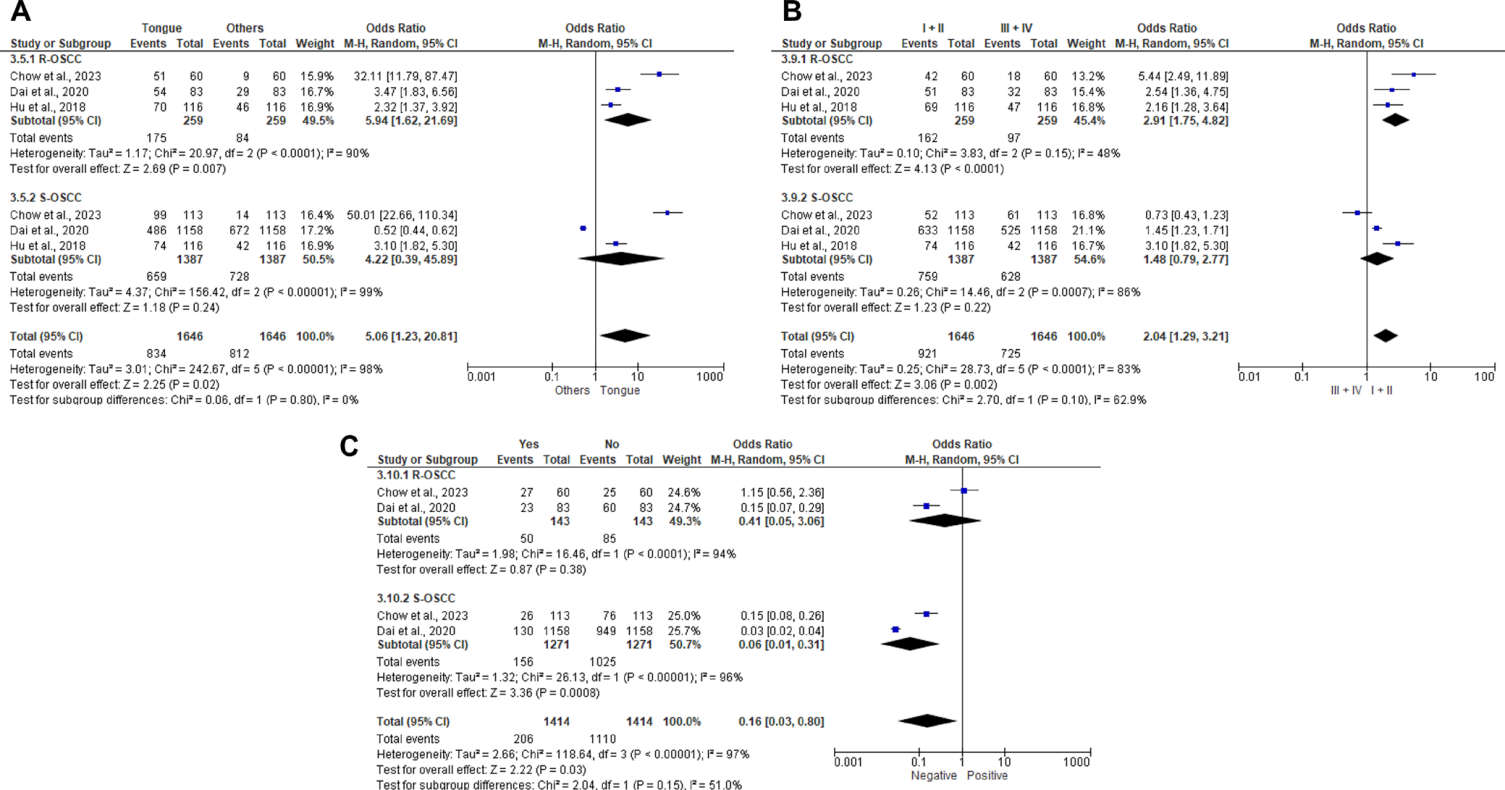
Pooled effect size of the association between R‐OSCC or S‐OSCC diagnosis and clinicopathological features. (A) Anatomic site. (B) Clinical stage. (C) Perineural invasion.

Four studies were included in the meta‐analysis; Toda et al. [23] was excluded due to lack of HR and insufficient data for OR calculation. The analysis showed a significant association between R‐OSCC and tongue tumors compared to other sites (OR: 5.94; 95% CI: 1.62–21.69; I^2^: 90%; *p* = 0.007), while no site differences were found in S‐OSCC (Figure 3A). Clinical stages I and II were also associated with R‐OSCC (OR: 2.91; 95% CI: 1.75–4.82; I^2^: 48%; *p* < 0.0001), with no stage differences in S‐OSCC (Figure 3B). Negative perineural invasion was linked to S‐OSCC (OR: 0.06; 95% CI: 0.01–0.31; I^2^: 96%; *p* = 0.0008), but not to R‐OSCC (Figure 3C). Other clinicopathological features were similar between groups (Figures S2 and S3).

**FIGURE 4:**
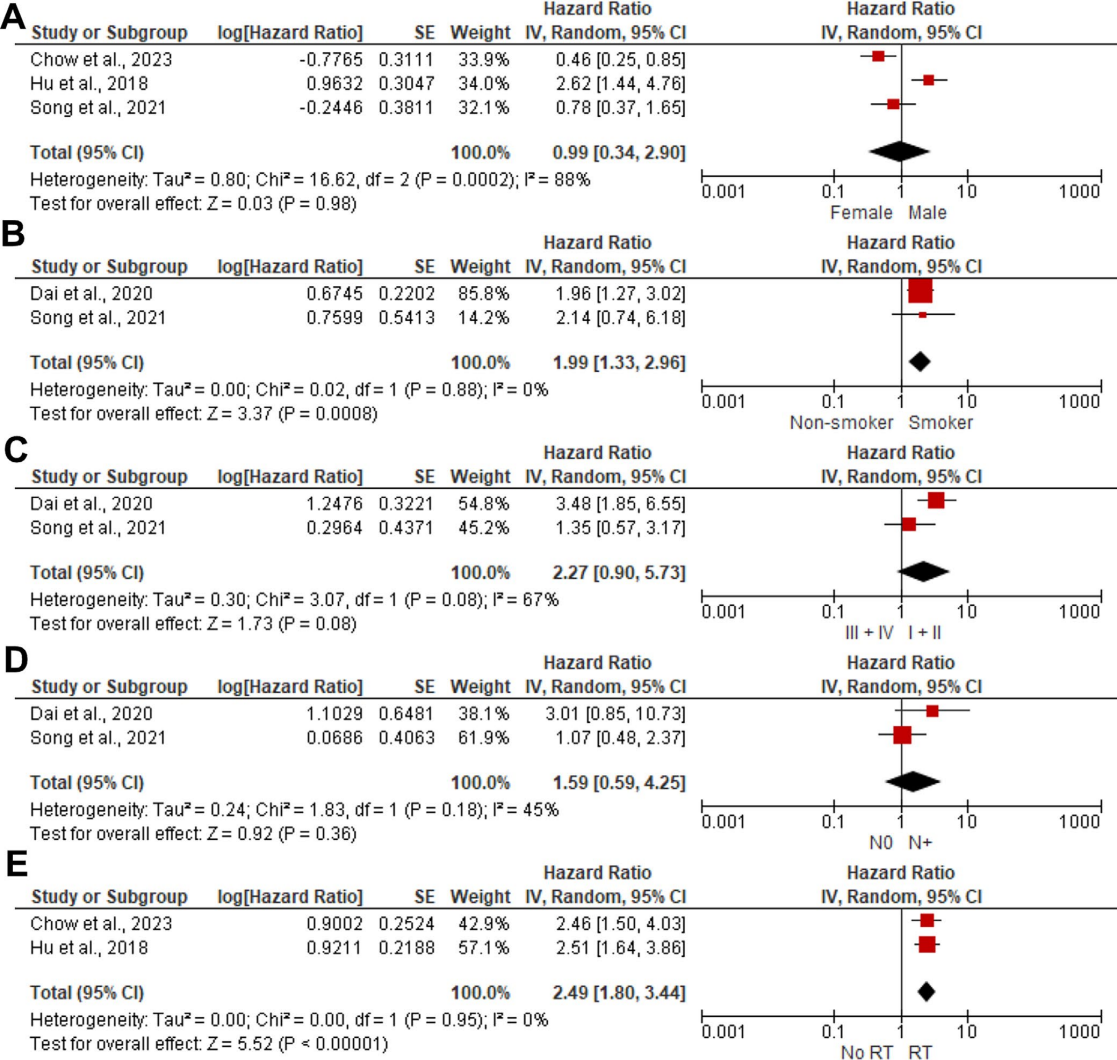
Pooled effect size of the risk factors for overall survival. (A) Sex. (B) Tobacco status. (C) Clinical stage. (D) Lymph node status. (E) Radiotherapy history.

**FIGURE 5:**
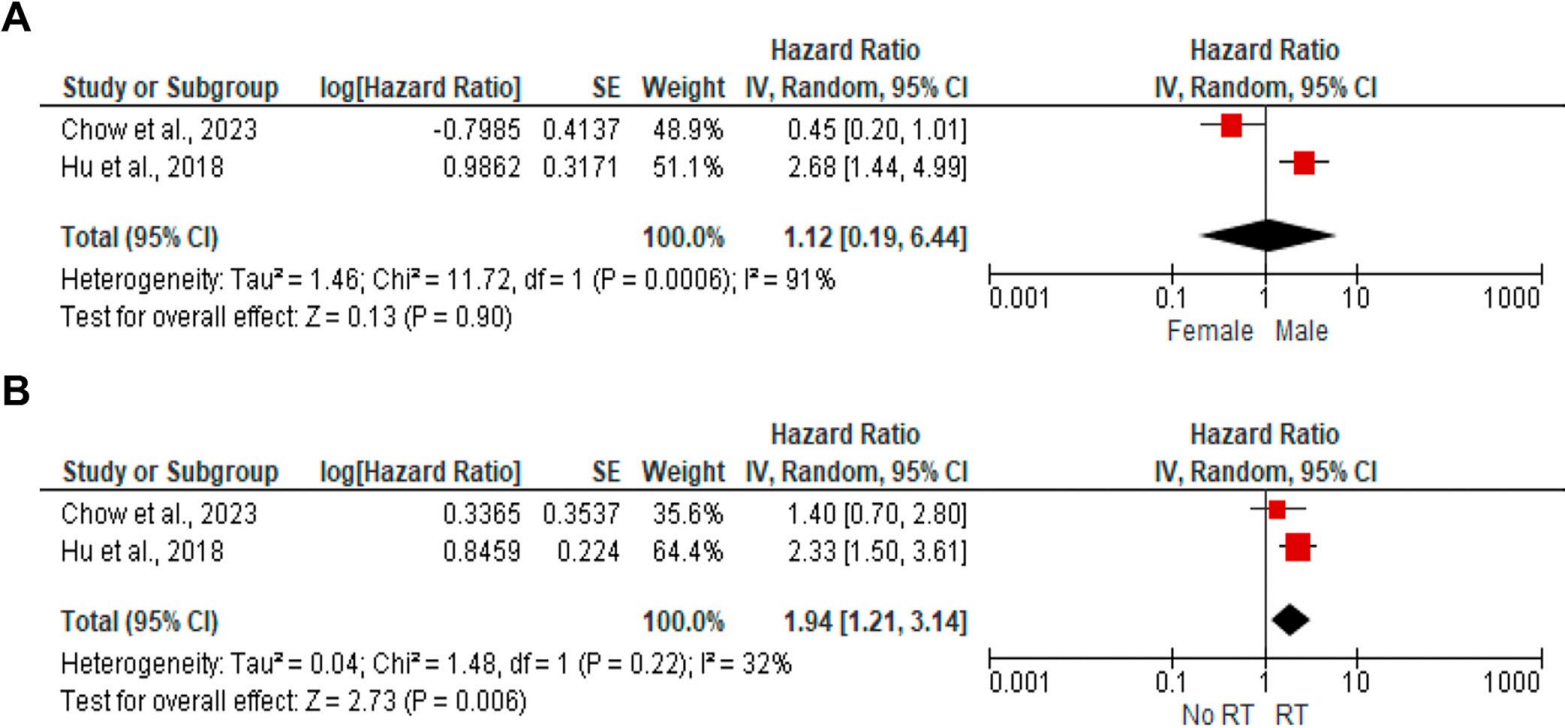
Pooled effect size of the risk factors for disease specific survival. (A) Sex. (B) Radiotherapy history.

The meta‐analysis identified smoking (HR: 1.99; 95% CI: 1.33–2.96; I^2^: 0%; *p* = 0.0008) and history of RT (HR: 2.49; 95% CI: 1.80–3.44; I^2^: 0%; *p* < 0.00001) as risk factors for OS in R‐OSCC patients. Sex, clinical stage, and lymph node status were not associated with OS (Figure 4). Additionally, prior RT was a risk factor for DSS (HR: 1.94; 95% CI: 1.21–3.14; I^2^: 32%; *p* = 0.006), while sex was not an independent risk factor for DSS (Figure 5).

## 4 Discussion

Information on radiation‐induced squamous cell carcinoma (R‐OSCC) is limited. This study reviewed the current literature to determine its clinicopathological profile and prognosis.

This review summarized data from 310 R‐OSCC patients in five studies, mostly from China (80%). Most primary tumors were nasopharyngeal carcinomas (NPC), common in China [22]. NPC patients have a 55% higher risk of second malignancies [27], and the oral cavity is often in the radiation field [22]. Radiation‐induced cancers tend to follow a pattern based on primary tumors; for example, breast cancer RT leads to secondary tumors in the lungs and esophagus [1]. NPC patients may have the highest R‐OSCC risk, highlighting the need for more studies in other countries and with different primary tumors.

Conventional RT was used in most R‐OSCC patients. However, this technique is not necessarily linked to a higher risk of radiation‐induced cancer. Intensity‐modulated RT redistributes the dose, shifting risk among tissues, but overall risk remains similar between techniques [28]. Patients received a mean radiation dose of 62.2 Gy, with R‐OSCC developing after an average latency of 142.1 months. For most of the included studies, loss to follow‐up was not explicitly addressed, and no strategies were reported to mitigate incomplete follow‐up, which may affect the accuracy of latency data. According to Berrington de Gonzalez et al. (2013) [29], the risk of radiation‐induced cancer increases 5‐ to 10‐fold at doses ≥ 40 Gy, which is below the doses observed here. A long latency period for R‐OSCC is well documented [6]. Similarly, in breast cancer patients, second cancer incidence rises over time, peaking at 10–15 years [30]. These findings highlight the need for prolonged follow‐up in head and neck RT patients to enable earlier R‐OSCC diagnosis.

Most patients with R‐OSCC were male, averaging 57.2 years, a demographic also common in S‐OSCC [31]. Tobacco (27.15% vs. 29.8%) and alcohol use (10.0% vs. 16.4%) rates were similar but slightly higher in S‐OSCC. Meta‐analysis showed comparable associations between diagnosis (R‐OSCC or S‐OSCC) and clinical factors such as sex, tobacco, and alcohol use. Thus, clinical characteristics are similar between R‐OSCC and S‐OSCC.

The tongue was the most affected site in the total sample and individual studies of R‐OSCC. An association between R‐OSCC diagnosis and tongue tumors was observed; however, no such association was found for S‐OSCC, despite the tongue being highly prevalent in S‐OSCC cases [31]. In R‐OSCC cases post‐RT for NPC, over 30% of lesions involved the dorsum—an uncommon site for S‐OSCC (< 10%) [13, 22]. Zhang et al. [22] noted that the posterior dorsum of the tongue is included in medium‐ and high‐dose radiation fields for NPC. This likely reflects the irradiation field, as NPC formed most of the sample.

Only R‐OSCC was associated with clinical stages I and II in the meta‐analysis. Patients with S‐OSCC showed a higher rate of lymph node metastases. Zhang et al. [22] and Sun et al. [13] compared tongue R‐OSCC and S‐OSCC, finding that R‐OSCC patients had fewer lymph node metastases and were diagnosed earlier, likely due to RT‐induced lymphatic atrophy and more frequent follow‐up, respectively. Additionally, negative perineural invasion was linked to S‐OSCC, suggesting that R‐OSCC can be invasive even at early stages. In fact, perineural invasion occurs independently of lymphatic invasion and represents an adverse prognostic factor in oral cancer, being associated with poorer overall survival, disease‐free survival, and locoregional recurrence [32]. More studies are needed to clarify its clinicopathological features.

Regarding prognostic factors, the pooled analysis showed that smoking history and prior RT increase mortality risk in R‐OSCC patients. Tobacco is a well‐known risk factor for oral squamous cell carcinoma [33]. A study comparing smokers and non‐smokers with oral cancer found higher all‐cause (*p* = 0.002) and cancer‐specific mortality (*p* = 0.010) in smokers [34]. The analysis also identified RT history as a risk factor for disease‐specific survival (DSS) in R‐OSCC. Hu et al. [17] confirmed this, while Chow et al. [24] did not. Dai et al. [26] reported lower DSS in R‐OSCC (62%) versus S‐OSCC (67%). Although not consistent across all studies, these data suggest R‐OSCC has poorer survival in malignancy‐specific deaths. Despite early clinical stages being associated with R‐OSCC, this was not a significant prognostic factor, indicating a more aggressive biology with prior RT as a key prognostic factor, unlike in S‐OSCC. Similarly, patients with radiation‐induced sarcoma had worse DSS than those with sporadic sarcoma [35].

### 4.1 Limitations

Some limitations should be noted. First, the small number of included studies limits generalizability. Second, most studies were conducted in China, reflecting its epidemiology. Third, nasopharyngeal carcinoma was the primary tumor in most studies, so radiation exposure was well defined, limiting applicability to other head and neck tumor sites. Additionally, some features could not be pooled due to inconsistent data presentation or assessment in only one study.

## 5 Conclusion

R‐OSCC predominantly affects males, with a mean age of 57.2 years, most commonly involving the tongue. It is mainly diagnosed at early stages, often without lymph node metastasis, but may present perineural invasion. Previous RT history is linked to shorter overall and disease‐specific survival.

## 6 Other Information

### 6.1 Protocol and Registration

The methods of this SR were established before starting the review, and the resulting protocol was based on PRISMA‐P (Preferred Reporting Items for Systematic Reviews and Meta‐analyses) [36] which was registered at the International Prospective Register of Systematic Reviews (PROSPERO) database under registration number CRD 42024560015 [37]. Additionally, the present SR was reported according to the Preferred Reporting Items for Systematic Reviews and Meta‐analyses (PRISMA) checklist [38].

## Funding

The authors have nothing to report.

## Conflicts of Interest

The authors declare no conflicts of interest.

## Supporting information


**Data S1:** Checklist Prisma_ROSCC.

**Figure S1:** Anatomic site distribution of radiation‐induced oral squamous cell carcinoma (R‐OSCC), created in https://BioRender.com.

**Figure S2:** Pooled effect size of the association between R‐OSCC or S‐OSCC diagnosis and clinical characteristics. (A) Sex. (B) Tobacco status. (C) Alcohol status.

**Figure S3:** Pooled effect size of the association between R‐OSCC or S‐OSCC diagnosis and pathological characteristics. (A) Tumor differentiation. (B) Tumor stage. (C) Lymph node status. (D) Lymphovascular invasion.

**Table S1:**—Search strategies used in each electronic database.

**Table S2:** Excluded articles and reasons for exclusion (*n* = 81).

**Table S3:** Clinical‐pathological characteristics of the sample with R‐OSCC of each included study in the SR.

**Table S4:** Summary of clinical‐pathological characteristics of the sample with s‐OSCC.

**Table S5:** jop70106‐sup‐0009‐TableS5.docx.**—**Risk of bias assessed using the Joanna Briggs Institute tool for use in Systematic Reviews. The risk of bias was categorized as high, when the study score up to 49% “yes”, moderate when the study scored 50% to 69% “yes”, and low when the study scored more than 70% “yes”.

## Data Availability

The data that support the findings of this study are available from the corresponding author upon reasonable request.
