# Supplementary figures and images for: Clinicopathological Analysis and Survival Outcomes of Radiation‐Induced Oral Squamous Cell Carcinoma: A Systematic Review and Meta‐Analysis
Source: J Oral Pathol Med. 2025 Dec 30;55(4):448–57. doi: 10.1111/jop.70106 (PMC13065901; doi:10.1111/jop.70106)

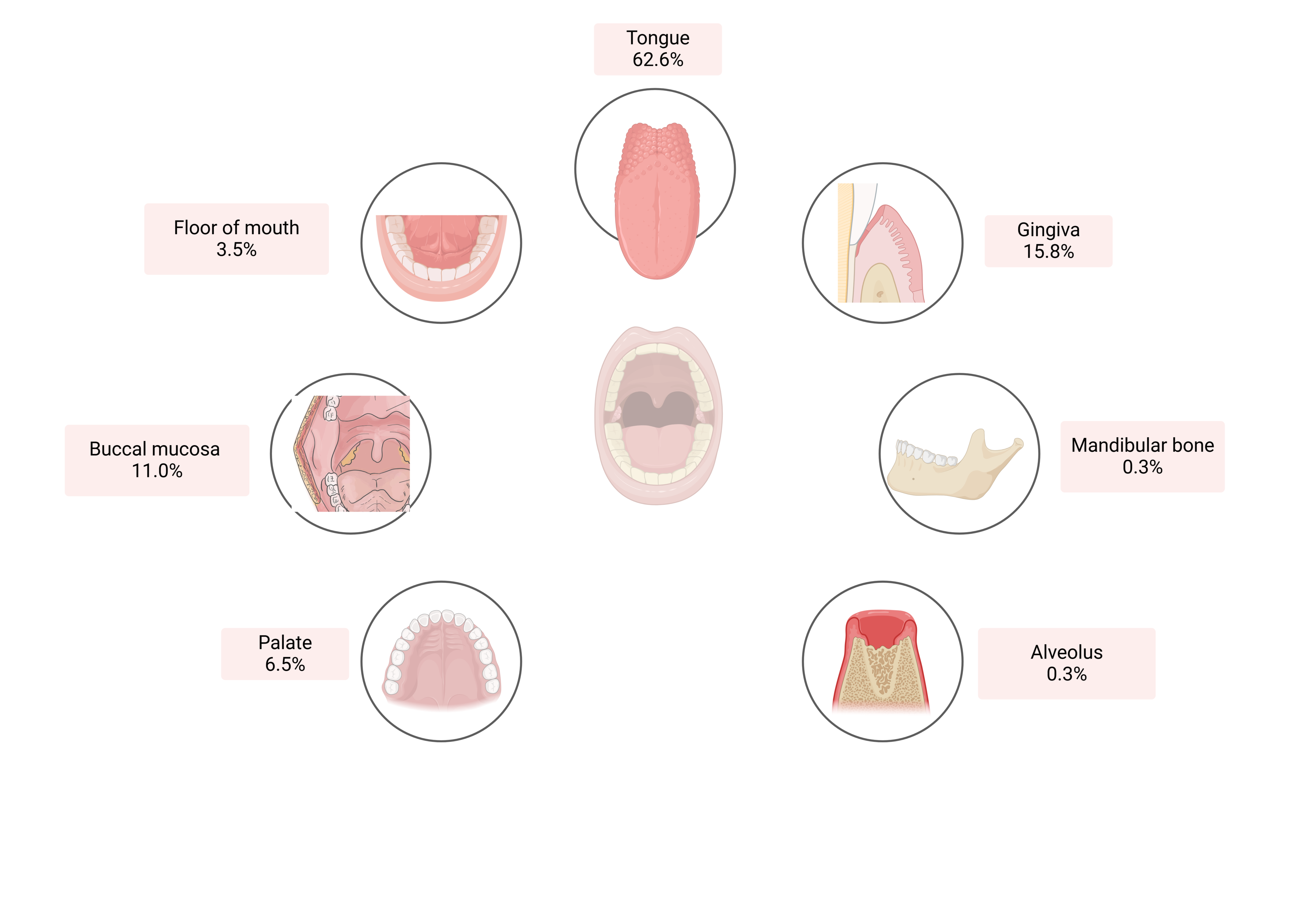

Supplement: Supplementary file 2 — Figure S1: Anatomic site distribution of radiation‐induced oral squamous cell carcinoma (R‐OSCC), created in https://BioRender.com. [file JOP-55-448-s006.tiff]

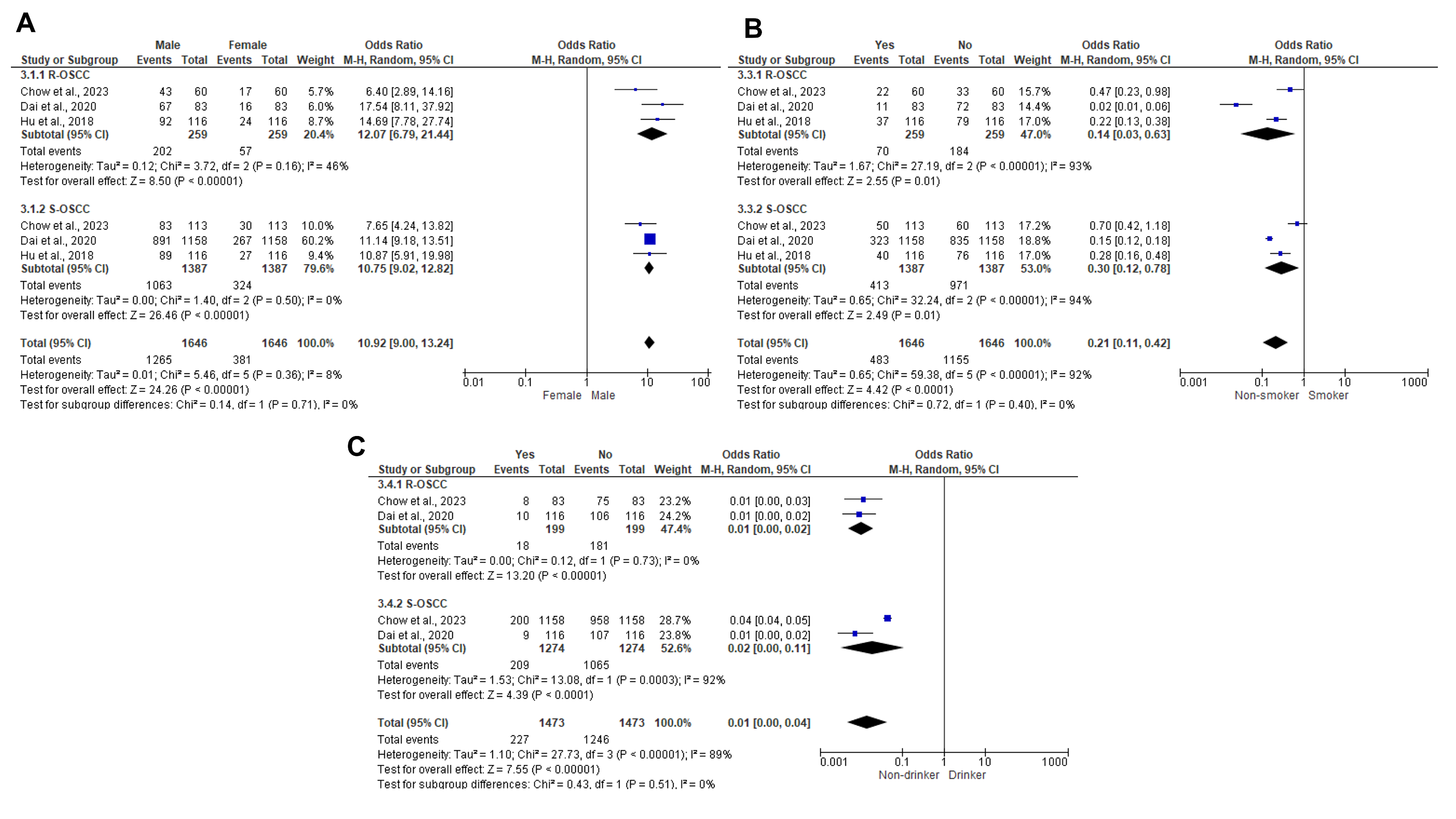

Supplement: Supplementary file 3 — Figure S2: Pooled effect size of the association between R‐OSCC or S‐OSCC diagnosis and clinical characteristics. (A) Sex. (B) Tobacco status. (C) Alcohol status. [file JOP-55-448-s005.tiff]

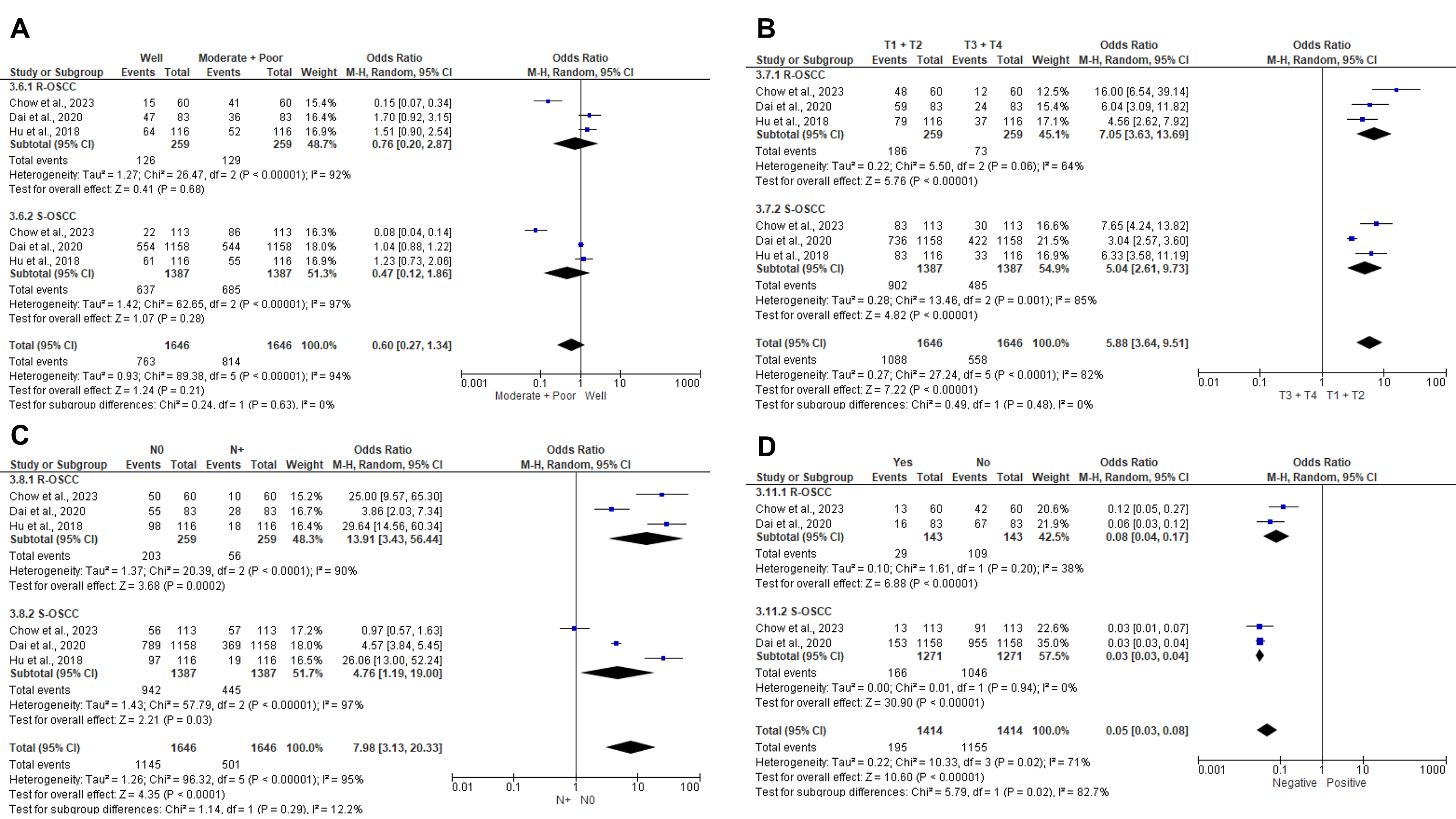

Supplement: Supplementary file 4 — Figure S3: Pooled effect size of the association between R‐OSCC or S‐OSCC diagnosis and pathological characteristics. (A) Tumor differentiation. (B) Tumor stage. (C) Lymph node status. (D) Lymphovascular invasion. [file JOP-55-448-s003.tiff]
