# Supplementary material for: Clinicopathological Analysis and Survival Outcomes of Radiation‐Induced Oral Squamous Cell Carcinoma: A Systematic Review and Meta‐Analysis
Source: J Oral Pathol Med. 2025 Dec 30;55(4):448–57. doi: 10.1111/jop.70106 (PMC13065901; doi:10.1111/jop.70106)
Supplement: Supplementary file 5 — Table S1:—Search strategies used in each electronic database. [file JOP-55-448-s009.docx]

**Supplementary Table 1** – Search strategies used in each electronic database.

| **Database** | **Search strategy**  (Search date: 10 July 2024, update 07 June 2025) | **Results** |
| --- | --- | --- |
| PubMed | (mouth[MeSH terms] OR mouth OR oral OR “buccal mucosa” OR “cheek mucosa” OR tongue OR “mouth floor” OR “floor of mouth” OR sublingual OR palate OR gingiva OR gingival OR gum* OR lip*) **AND** (Carcinoma, Squamous Cell[MeSH terms] OR “Squamous Cell Carcinoma*” OR “Squamous Carcinoma*” OR “Epidermoid Carcinoma*” OR “Planocellular Carcinoma*”) **AND** (Post-radiotherapy OR Post-radiation OR Postirradiation OR Postradiotherapy OR Radiation-induced OR Radiotherapy-induced OR radio-induced OR radiation-associated OR radiotherapy-associated OR radio-associated OR “after radiotherapy” OR “after radiation” OR “history of radiotherapy” OR “history of radiation” OR “adjuvant radiotherapy”) | 1870 |
| Scopus | TITLE-ABS-KEY(mouth OR oral OR “buccal mucosa” OR “cheek mucosa” OR tongue OR “mouth floor” OR “floor of mouth” OR sublingual OR palate OR gingiva OR gingivas OR gingival OR gum OR gums OR lip OR lips) AND TITLE-ABS-KEY(Carcinoma, Squamous Cell OR “Squamous Cell Carcinomas” OR “Squamous Cell Carcinoma” OR “Squamous Carcinoma” OR “Squamous Carcinomas” OR “Epidermoid Carcinoma” OR “Epidermoid Carcinomas” OR “Planocellular Carcinoma” OR “Planocellular Carcinomas”) AND TITLE-ABS-KEY(Post-radiotherapy OR Post-radiation OR Postirradiation OR Postradiotherapy OR Radiation-induced OR Radiotherapy-induced OR radio-induced OR radiation-associated OR radiotherapy-associated OR radio-associated OR “after radiotherapy” OR “after radiation” OR “history of radiotherapy” OR “history of radiation” OR “adjuvant radiotherapy”) | 2096 |
| Embase | ('mouth'/de OR oral OR 'buccal mucosa'/de OR 'cheek mucosa'/de OR 'tongue'/de OR 'mouth floor'/de OR 'floor of mouth' OR sublingual OR 'palate'/de OR 'gingiva'/de OR gingivas OR gingival OR 'gum'/de OR gums OR 'lip'/de OR 'lips'/de) AND ('carcinoma,'/de AND squamous AND 'cell'/de OR 'squamous cell carcinomas' OR 'squamous cell carcinoma'/de OR 'squamous carcinoma'/de OR 'squamous carcinomas' OR 'epidermoid carcinoma'/de OR 'epidermoid carcinomas' OR 'planocellular carcinoma'/de OR 'planocellular carcinomas') AND ('post radiotherapy' OR 'post radiation' OR postirradiation OR postradiotherapy OR 'radiation induced' OR 'radiotherapy induced' OR 'radio induced' OR 'radiation associated' OR 'radiotherapy associated' OR 'radio associated' OR 'after radiotherapy' OR 'after radiation' OR 'history of radiotherapy'/de OR 'history of radiation' OR 'adjuvant radiotherapy'/de) | 769 |
| Web of Science | TS=(mouth OR oral OR “buccal mucosa” OR “cheek mucosa” OR tongue OR “mouth floor” OR “floor of mouth” OR sublingual OR palate OR gingiva OR gingivas OR gingival OR gum OR gums OR lip OR lips) AND TS=(Carcinoma, Squamous Cell OR “Squamous Cell Carcinomas” OR “Squamous Cell Carcinoma” OR “Squamous Carcinoma” OR “Squamous Carcinomas” OR “Epidermoid Carcinoma” OR “Epidermoid Carcinomas” OR “Planocellular Carcinoma” OR “Planocellular Carcinomas”) AND TS=(Post-radiotherapy OR Post-radiation OR Postirradiation OR Postradiotherapy OR Radiation-induced OR Radiotherapy-induced OR radio-induced OR radiation-associated OR radiotherapy-associated OR radio-associated OR “after radiotherapy” OR “after radiation” OR “history of radiotherapy” OR “history of radiation” OR “adjuvant radiotherapy”) | 1145 |
| Lilacs | (boca OR mouth OR oral) AND (“Carcinoma de células escamosas” OR “squamous cells carcinoma” OR “carcinoma espinocelular”) AND (“pós radioterapia” OR Post-Radiotherapy) | 19 |
| Google Scholar | (mouth OR oral) AND (“squamous cells carcinoma” OR “epidermoid carcinoma” OR ) AND (Post-Radiotherapy OR Post-radiation OR Postirradiation OR Postradiotherapy) | 100 |
