# Supplementary material for: Clinicopathological Analysis and Survival Outcomes of Radiation‐Induced Oral Squamous Cell Carcinoma: A Systematic Review and Meta‐Analysis
Source: J Oral Pathol Med. 2025 Dec 30;55(4):448–57. doi: 10.1111/jop.70106 (PMC13065901; doi:10.1111/jop.70106)
Supplement: Supplementary file 6 — Table S2: Excluded articles and reasons for exclusion (n = 81). [file JOP-55-448-s008.docx]

**Supplementary Table 2**. Excluded articles and reasons for exclusion (n=81).

|  | **Articles** | **Reason for exclusion** |
| --- | --- | --- |
| 1 | Abrigo JM, King AD, Leung SF, et al. MRI of radiation-induced tumors of the head and neck in post-radiation nasopharyngeal carcinoma. Eur Radiol. 2009;19(5):1197-1205. doi:10.1007/s00330-008-1265-6 | 1 |
| 2 | Amemiya K, Shibuya H, Yoshimura R, Okada N. The risk of radiation-induced cancer in patients with squamous cell carcinoma of the head and neck and its results of treatment. Br J Radiol. 2005;78(935):1028-1033. doi:10.1259/bjr/86352309 | 1 |
| 3 | Brandão TB, Morais-Faria K, Ribeiro ACP, Rivera C, Salvajoli JV, Lopes MA et al. Locally advanced oral squamous cell carcinoma patients treated with photobiomodulation for prevention of oral mucositis: retrospective outcomes and safety analyses. Support Care Cancer. 2018;26(7):2417-2423. doi:10.1007/s00520-018-4046-z | 1 |
| 4 | Buchberger AMS, Strzelczyk EA, Wollenberg B, Combs SE, Pickhard A, Pigorsch SU. Report on Late Toxicity in Head-and-Neck Tumor Patients with Long Term Survival after Radiochemotherapy. Cancers (Basel). 2021;13(17):4292. Published 2021 Aug 26. doi:10.3390/cancers13174292 | 1 |
| 5 | Byers RM, O'Brien J, Waxler J. The therapeutic and prognostic implications of nerve invasion in cancer of the lower lip. Int J Radiat Oncol Biol Phys. 1978;4(3-4):215-217. doi:10.1016/0360-3016(78)90140-2 | 1 |
| 6 | Chen YC, Wang YP, Hsieh MS, Chang JY. Mucoepidermoid carcinoma arising from a glandular odontogenic cyst of posterior maxilla and further development into a radiation-induced second primary squamous cell carcinoma. J Dent Sci. 2024;19(1):675-677. doi:10.1016/j.jds.2023.10.002 | 4 |
| 7 | Chow JH, Cheuk W, Au DWY, Wong CF, Tam AHP, Cheung KM, Cho WCS. 225MO Clinical, pathologic and immune-related features of radiation-associated oral cavity squamous cell carcinoma: A matched cohort study. Annals of Oncology. 2022;33:S1523. | 4 |
| 8 | Chua DT, Tian Y, Wei WI. Late oral complications following radiotherapy for head and neck cancers. Expert Rev Anticancer Ther. 2007 Sep;7(9):1215-24. doi: 10.1586/14737140.7.9.1215. PMID: 17892422. | 4 |
| 9 | Colbert S, Algholmy M, Gray M, Walji S, Davies J. Management of the cervical lymph node metastasis of unknown origin. British Journal of Oral and Maxillofacial Surgery. 2011: 49, S43. | 4 |
| 10 | Cooney TR, Poulsen MG. Is routine follow-up useful after combined-modality therapy for advanced head and neck cancer?. Arch Otolaryngol Head Neck Surg. 1999;125(4):379-382. doi:10.1001/archotol.125.4.379 | 1 |
| 11 | Coppen C, de Wilde PC, Pop LA, van den Hoogen FJ, Merkx MA. Treatment results of patients with a squamous cell carcinoma of the buccal mucosa. Oral Oncol. 2006;42(8):795-799. doi:10.1016/j.oraloncology.2005.11.017 | 1 |
| 12 | Cremers CW, De Mönnink JP, Arts N, Joosten FB, Kremer H, Hoefsloot L. Clinical report on the L95P mutation in a Dutch family with paraganglioma. Otol Neurotol. 2002;23(5):755-759. doi:10.1097/00129492-200209000-00024 | 1 |
| **13** | De Biasio MJ, Mohan R, Hendler A, Tsai, CJ, McPartlin A, Hosni et al. Lymphatic mapping in second primary or recurrent oral cavity cancer with prior neck treatment: A case series and scoping review. Oral Oncology.2024:159, 107077. | 3 |
| 14 | Dombrowski ND, Wolter NE, Irace AL, et al. Squamous cell carcinoma of the head and neck in children. Int J Pediatr Otorhinolaryngol. 2019;117:131-137. doi:10.1016/j.ijporl.2018.11.019 | 1 |
| 15 | Elicin O, Sermaxhaj B, Bojaxhiu B, et al. Incidence of second primary cancers after radiotherapy combined with platinum and/or cetuximab in head and neck cancer patients. Inzidenz von metachronen Zweitkarzinomen nach Strahlentherapie in Kombination mit Platin und/oder Cetuximab bei Patienten mit Kopf-Hals-Tumoren. Strahlenther Onkol. 2019;195(6):468-474. doi:10.1007/s00066-018-1400-5 | 3 |
| 16 | Levi F, Randimbison L, Vecchia CL, Erler G, Te, V. C. Incidence of invasive cancers following squamous cell skin cancer. American journal of epidemiology. 1997:146(9), 734-739. | 1 |
| 17 | Fallai C, Bolner A, Signor M, et al. Long-term results of conventional radiotherapy versus accelerated hyperfractionated radiotherapy versus concomitant radiotherapy and chemotherapy in locoregionally advanced carcinoma of the oropharynx. Tumori. 2006;92(1):41-54. doi:10.1177/030089160609200108 | 1 |
| 18 | Farhadieh RD, Otahal P, Rees CG, Salardini A, Russell P, Smee R. Radiotherapy is not associated with an increased rate of Second Primary Tumours in Oral Squamous Carcinoma: a study of 370 patients. Oral Oncol. 2009;45(11):941-945. doi:10.1016/j.oraloncology.2009.05.634 | 3 |
| 19 | Fu X, Chen S, Chen W, et al. Clinical analysis of second primary gingival squamous cell carcinoma after radiotherapy. Oral Oncol. 2018;84:20-24. doi:10.1016/j.oraloncology.2018.06.018 | 7 |
| 20 | Fujisawa R, Shibuya H, Harata N, Yuasa-Nakagawa K, Toda K, Hayashi K. Chronological shifts and changing causes of death after radiotherapy for early-stage oral cancer. Int J Clin Oncol. 2014;19(1):24-29. doi:10.1007/s10147-013-0519-8 | 3 |
| 21 | Gates M, Hughes J, Link. Wound and Radiation Complications in Craniofacial Resection of Sinonasal Tumors. Journal of Neurological Surgery Part B: Skull Base. 2017 | 4 |
| 22 | González-García R, Naval-Gías L, Román-Romero L, Sastre-Pérez J, Rodríguez-Campo FJ. Local recurrences and second primary tumors from squamous cell carcinoma of the oral cavity: a retrospective analytic study of 500 patients. Head Neck. 2009;31(9):1168-1180. doi:10.1002/hed.21088 | 1 |
| 23 | Grant DG, Salassa JR, Hinni ML, Pearson BW, Hayden RE, Perry WC. Transoral laser microsurgery for recurrent laryngeal and pharyngeal cancer. Otolaryngol Head Neck Surg. 2008;138(5):606-613. doi:10.1016/j.otohns.2007.12.046 | 1 |
| 24 | Grant DG, Salassa JR, Hinni ML, Pearson BW, Perry WC. Carcinoma of the tongue base treated by transoral laser microsurgery, part two: Persistent, recurrent and second primary tumors. Laryngoscope. 2006;116(12):2156-2161. doi:10.1097/01.mlg.0000244176.74302.e6 | 3 |
| 25 | Harada, T., Nameki, H., Kano, S., & Okuno, T. (1994). A case of double cancer induced by irradiation for head and neck hemangioma. Jibi Inkoka Tenbo;(Japan), 37(4). | 6 |
| 26 | Hashibe M, Ritz B, Le AD, Li G, Sankaranarayanan R, Zhang ZF. Radiotherapy for oral cancer as a risk factor for second primary cancers. Cancer Lett. 2005;220(2):185-195. doi:10.1016/j.canlet.2004.10.023 | 3 |
| 27 | Ho MW, Field EA, Field JK, Risk JM, Rajlawat BP, Rogers SN, Shaw RJ. Outcomes of oral squamous cell carcinoma arising from oral epithelial dysplasia: rationale for monitoring premalignant oral lesions in a multidisciplinary clinic. Br J Oral Maxillofac Surg. 2013;51(7):594-599. doi:10.1016/j.bjoms.2013.03.014 | 1 |
| 28 | Horiuchi J, Okuyama T, Shibuya H, Takeda M. Gan No Rinsho. 1984;30(15):1855-1860. | 6 |
| 29 | Horiuchi J, Shibuya H, Takeda M, Takagi M. Secondary and multiple primary cancers relating radiation therapy for cancer of the oral region. Nippon Gan Chiryo Gakkai-Shi. 1985;20(3). | 5 |
| 30 | Hunter WP, Mah-Ginn K, Zhang F, Faquin W, August M, Peacock ZS. Risk Factors for Malignant Transformation of Squamous Intraepithelial Lesions. Journal of Oral and Maxillofacial Surgery. 2020;78(10):e30. | 4 |
| 31 | Ikarashi F, Nonomura N, NakanoY. Clinical study on laryngeal cancer. Nippon Jibiinkoka Gakkai Kaiho. 1990; 93(5): 772-778. | 6 |
| 32 | Kim BY, Cho KR, Sohn JH, Kim JY. Sarcomatoid carcinoma after radiotherapy for early-stage oral squamous cell carcinoma: Case report. Medicine (Baltimore). 2019;98(27):e16003. | 1 |
| 33 | Ko HH, Cheng SL, Lee JJ, Chen HM, Wang CW, Cheng SJ,et al. Factors influencing the incidence and prognosis of second primary tumors in patients with oral squamous cell carcinoma. Head Neck. 2016;38(10):1459-1466. doi:10.1002/hed.24457 | 1 |
| 34 | Kuhnt T, Klockenbrink U, Pelz T, Wienke A, Janich M, Sandner A, Schubert J. Postoperative radiotherapy versus concurrent radiochemotherapy for locally advanced squamous cell carcinoma of the oral cavity and the oropharynx. a retrospective analysis of high-risk patients. New Armenian Medical Journal. 2009;3(3):11-21. | 1 |
| 35 | Kwon M, Lee JI, Roh JL, et al. Second cancer incidence and risk factors in patients with salivary gland cancers. JAMA Otolaryngol Head Neck Surg. 2014;140(2):118-123. doi:10.1001/jamaoto.2013.6149 | 2 |
| 36 | Levin RJ, Mahne JP, Huang MY, Fedok FG.  Unusual second primary malignancies after successful treatment of nasopharyngeal carcinoma.  Skull Base Surgery. 1990 | 4 |
| 37 | Lin GC.Postradiation cancer of oral and maxillofacial regions. Zhonghua Zhong Liu Za Zhi. 1990;12(4):301-303. | 6 |
| 38 | Liu C, Liao L, Wu G, et al. Radiation-induced second primary squamous cell carcinoma of the oral cavity after radiotherapy for nasopharyngeal carcinoma. Oral Oncol. Published online June 27, 2020. doi:10.1016/j.oraloncology.2020.104863 | 7 |
| 39 | Maeda H, Yoshida J, Sasaki R, Matsunaga T. Multiple primary cancers in patients with oropharyngeal neoplasms. Practica Oto-Rhino-Laryngologica. 1985;78(8):1603–1608. | 6 |
| 40 | Maruyama N, Sasaki T, Arasaki A, Matsuzaki A, Nakasone T, Teruya T et al. Thymoma appearing 9 years after the resection of squamous cell carcinoma of the lip: A case report of triple primary tumors and literature review. Oncology Letters. 2019 | 1 |
| 41 | Mendenhall NP, Shuster JJ, Million RR. The impact of stage and treatment modality on the likelihood of second malignancies and hematopoietic disorders in Hodgkin's disease. Radiotherapy and Oncology. 1989;14(3):219-229. | 1 |
| 42 | Min SK, Choi SW, Lim J, Park JY, Jung KW, Won YJ. Second primary cancers in patients with oral cavity cancer included in the Korea Central Cancer Registry. Oral Oncol. 2019;95:16-28. doi:10.1016/j.oraloncology.2019.05.025 | 1 |
| 43 | Miyahara H, Matsuoka H, Ohbuchi M, Tohya M, Sato T, Maeda K. Radiation cancers in the head and neck region. Nippon Jibiinkoka Gakkai Kaiho. 1978;81(3):233–241. doi:10.3950/jibiinkoka.81.23 | 6 |
| 44 | Mohammadi K, Mohiyuddin SMA, Harshitha N, Suresh TN, Prasad CSBR, Sagayaraj A, Deo RP, Gopinath KS, Manjunath GN, Prashanth Babu A, Krishna P, Abhilasha K, Brindha HS, Aishwarya Raj Pillai D, Gupta A. Outcome of Treatment in Verrucous Carcinoma of Oral Cavity: A Tertiary Rural Hospital Experience. Indian J Otolaryngol Head Neck Surg. 2022 Oct;74(Suppl 2):1768-1772. doi: 10.1007/s12070-019-01782-z. Epub 2020 Jan 2. PMID: 36452610; PMCID: PMC9702320. | 1 |
| 45 | Monzen Y, Hayashi K, Sasano T, Ikenaga K. Radiation induced cancer after hemangioma of the tongue. Jibi Inkoka Tokeibu Geka. 1990;62(10: 871-873. | 6 |
| 46 | Nariai Y, Kanno T, Sekine J.Histopathological features of secondary squamous cell carcinoma around a dental implant in the mandible after chemoradiotherapy: a case report with a clinicopathological review. Journal of Oral and Maxillofacial Surgery.2016;74(5): 982-990. | 1 |
| 47 | Ng E, Bae YSC, Meehan S, Geronemus RG. Keratoacanthoma arising within a port wine stain. Journal of the American Academy of Dermatology. 2015;72(5): AB261-AB261 | 4 |
| 48 | Ono I, Ebihara S, Egawa S, Akine Y. The second carcinoma of the anterior two-thirds of the tongue after successful radiotherapy to the tongue. Int J Radiat Oncol Biol Phys. 1989;(4):773-8. | 1 |
| 49 | Osano H, Watanabe S, Numao A. [A case report of radiation-induced cancer of the buccal mucosa, effectively treated with CF therapy]. Gan To Kagaku Ryoho. 200;28(2):257-60. | 6 |
| 50 | Pandya JA, Srikant N, Boaz K, Manaktala N, Kapila SN, Yinti SR. Post-radiation changes in oral tissues - An analysis of cancer irradiation cases. South Asian J Cancer. 201;3(3):159-62. doi: 10.4103/2278-330X.136785. | 1 |
| 51 | Papadimitrakopoulou VA, Dimery IW, Lee JJ, Perez C, Hong WK, Lippman SM. Cisplatin, fluorouracil, and L-leucovorin induction chemotherapy for locally advanced head and neck cancer: the M.D. Anderson Cancer Center experience. Cancer J Sci Am. 1997;3(2):92-9. | 5 |
| 52 | Perez CA, Kraus FT, Evans JC, Powers WE. Anaplastic transformation in verrucous carcinoma of the oral cavity after radiation therapy. Radiology. 1966;86(1):108-15. doi: 10.1148/86.1.108. | 1 |
| 53 | Pialat PM, Mounie M, Podeur F, Gassa F, Suchaud JP, Fleury B, Racadot S, Serre AA, Pommier P. Salvage brachytherapy with or without external beam radiotherapy for oral or oropharyngeal squamous cell carcinomas in previously irradiated areas: carcinologic and toxicity outcomes of 25 patients. J Contemp Brachytherapy. 2021;13(4):402-409. | 3 |
| 54 | Point D, Rodriguez J, Ferrante B, Brugère J. Cancers du voile du palais. Résultats de la chirurgie de rattrapage [Cancers of the soft palate. Results of repeat surgery]. Ann Otolaryngol Chir Cervicofac. 1987;104(6):395-7. | 6 |
| 55 | Pradhan SA, Pai PS, Neeli SI, D'Cruz AK. Transoral laser surgery for early glottic cancers. Arch Otolaryngol Head Neck Surg. 2003;129(6):623-5. | 2 |
| 56 | Rennemo E, Zätterström U, Evensen J, Boysen M. Reduced risk of head and neck second primary tumors after radiotherapy. Radiother Oncol. 2009;93(3):559-62. doi: 10.1016/j.radonc.2009.08.005. | 1 |
| 57 | Sakamoto S, Ohno T, Miyazaki T, Chiyoda T,Sato. Two Cases of Squamous Cell Carcinoma Arising in Free Flaps after Oral Reconstruction. Practica oto-rhino-laryngologica.(2015):141, 64-65. | 6 |
| 58 | Shingaki S, Kobayashi T, Suzuki I, Kohno M, Nakajima T. Surgical treatment of stage I and II oral squamous cell carcinomas: analysis of causes of failure. Br J Oral Maxillofac Surg. 1995;33(5):304-8. doi: | 1 |
| 59 | Siar CH, Ng KH. Adenosquamous carcinoma of the floor of the mouth and lower alveolus: a radiation-induced lesion? Oral Surg Oral Med Oral Pathol. 1987;63(2):216-20. doi: 10.1016/0030-4220(87)90315-x. | 1 |
| **60** | Slater NN, Farsi S, Rogers AL, Herberger L, Penagaricano J, McKee S, Moreno MA. Reirradiation in head and neck squamous cell carcinoma; prognostic indicators, oncologic and functional outcomes. American Journal of Otolaryngology. (2024):45(6), 104482. | 3 |
| 61 | Spencer SA, Harris J, Wheeler RH, Machtay M, Schultz C, Spanos W, Rotman M, Meredith R, Ang KK. Final report of RTOG 9610, a multi-institutional trial of reirradiation and chemotherapy for unresectable recurrent squamous cell carcinoma of the head and neck. Head Neck. 2008;30(3):281-8. | 1 |
| 62 | Steneker M, Lomax A, Schneider U. Intensity modulated photon and proton therapy for the treatment of head and neck tumors. Radiother Oncol. 2006;80(2):263-7. doi: 10.1016/j.radonc.2006.07.025. | 1 |
| 63 | Sumitsawan Y, Chaiyasate S, Chitapanarux I, Anansuthiwara M, Roongrotwattanasiri K, Vaseenon V, Tooncam H. Late complications of radiotherapy for nasopharyngeal carcinoma. Auris Nasus Larynx. 2009;36(2):205-9. doi: 10.1016/j.anl.2008.04.006. | 1 |
| 64 | Sun C, Hu Z, Zhong Z, Jiang Y, Sun R, Fei J, Xi Y, Li X, Song M, Li W, Li Q. Clinical and prognostic analysis of second primary squamous cell carcinoma of the tongue after radiotherapy for nasopharyngeal carcinoma. Br J Oral Maxillofac Surg. 2014;52(8):715-20. doi: 10.1016/j.bjoms.2014.06.015. | 7 |
| 65 | Sun C, Wang X, Zhong Z, et al. Differences in Clinicopathological Characteristics and Prognosis Between Primary and Postirradiation Tongue Squamous Cell Carcinoma. J Oral Maxillofac Surg. 2017;75(10):2235-2241. doi:10.1016/j.joms.2017.02.020 | 7 |
| 66 | Takahashi H, Watanabe K, Shirakura M, Honkura Y, Yamauchi D, Katori. Five cases of spindle cell carcinoma of the head and neck region. Practica Otologica. 2018;111(8):557-563. | 5 |
| 67 | Tanaka N, Hsieh KJ, Sasaki T, Tomizuka K, Hashimoto K, Shioda S. Squamous cell carcinoma after radiotherapy for cheek papilloma. Nippon Rinsho Denshi Kenbikyo Gakkai-Shi. 1989;22(4):409-414. | 5 |
| 68 | Tirelli G, Piovesana M, Bonini P, Gatto A, Azzarello G, Boscolo Nata F. Follow-up of oral and oropharyngeal cancer using narrow-band imaging and high-definition television with rigid endoscope to obtain an early diagnosis of second primary tumors: a prospective study. Eur Arch Otorhinolaryngol. 2017;274(6):2529-2536. . | 1 |
| 69 | Tishbejn AV; Yampol'skaya SA. Development of malignant neoplasms after repeated radiotherapy. Voprosy Onkologii. 1975;21(2):103-104. | 6 |
| 70 | Two Cases of Tongue Cancer Reappeared in the Irradiated Field More than 20 Years After Brachytherapy | 6 |
| 71 | Wang JN, Li RY, Cui MY. [Radiation-induced maxillary malignancies: complications of radiotherapy treatment for head-neck malignant tumors]. Zhonghua Kou Qiang Yi Xue Za Zhi. 2008 Dec;43(12):713-5. Chinese. PMID: 19134344. | 6 |
| 72 | Wang L, Wang S, Zhang J, Peng J, Cheng B, Li H, Hu Q. Radiotherapy upregulated immune checkpoints contribute to the development of second primary OSCC. Oral Dis. 2024;30(4):2188-2201 | 7 |
| 73 | Wang H, Liu M, He L, Xi M, Liu H, Hu Y, Cai L. Malignancies induced by radiation therapy for nasopharyngeal carcinoma-A report of 198 cases. Chinese Journal of Clinical Oncology. 2008;35(6):313-316. | 6 |
| 74 | Wolff KD, Rau A, Ferencz J, Langer T, Kesting M, Nieberler M, Wesselmann S. Effect of an evidence-based guideline on the treatment of maxillofacial cancer: A prospective analysis. J Craniomaxillofac Surg. 2017;45(3):427-431. | 1 |
| 75 | Wutzl A, Ploder O, Kermer C, Millesi W, Ewers R, Klug C. Mortality and causes of death after multimodality treatment for advanced oral and oropharyngeal cancer. J Oral Maxillofac Surg. 2007 Feb;65(2):255-60. doi: 10.1016/j.joms.2006.09.007. PMID: 17236930. | 1 |
| 76 | Zhang P, Zhang L, Liu H, Zhao L, Li Y, Shen JX et al.Clinicopathologic Characteristics and Prognosis of Tongue Squamous Cell Carcinoma in Patients with and without a History of Radiation for Nasopharyngeal Carcinoma: A Matched Case-Control Study. Cancer Res Treat. 2017;49(3):695-705. | 7 |
| 77 | Zhen W, Lydiatt WM, Ganti AK, Lydiatt DD, Richards AT, Enke CA. Treatment Outcomes of Squamous Cell Carcinoma of the Base of Tongue Treated with Primary IMRT with or without Chemotherapy. International Journal of Radiation Oncology, Biology, Physics. 2010;78(3):S474. | 4 |
| 78 | Zhu G, Chen Y, Zhu Z, Lu L, Bi X, Deng Q, Chen X, Su H, Liu Y, Guo H, Zheng T, Yu H, Zhang Y. Risk of second primary cancer after treatment for esophageal cancer: a pooled analysis of nine cancer registries. Dis Esophagus. 2012;25(6):505-11. doi: 10.1111/j.1442-2050.2011.01273. | 3 |
| 79 | Zhu X, Zhou J, Zhou L, Zhang M, Gao C, Tao L. Association between postoperative radiotherapy for young-onset head and neck cancer and long-term risk of second primary malignancy: a population-based study. J Transl Med. 2022 Sep 5;20(1):405. doi: 10.1186/s12967-022-03544-y. PMID: 36064552; PMCID: PMC9446763. | 1 |
| 80 | Zhuang S, Guo Z, Yang A, Zhang Q, Chen S, Wang L, Song M.Clinical analysis of second primary oral squamous cancer in patients with nasopharyngeal carcinoma after radiotherapy. Chinese Journal of Clinical Oncology. 2011;38(17):1032-1034. | 6 |
| 81 | Zwetyenga N, Majoufre-Lefebvre C, Siberchicot F, Demeaux H, Pinsolle J. Les carcinomes épidermoïdes de la langue. Résultats du traitement et pronostic [Squamous-cell carcinoma of the tongue: treatment results and prognosis]. Rev Stomatol Chir Maxillofac. 2003 Feb;104(1):10-7. French. PMID: 12644785. | 6 |

Legends:

1. Studies that did not evaluate the clinicopathological features of radiation-

induced oral SCC;

2. Studies that assessed patients with radiation-induced SCC at anatomical sites other oral

cavity;

3. Studies in which data on the clinicopathological characteristics of radiation-

induced oral SCC were not available for data extraction due to grouping with

non-radio-induced oral SCC carcinoma or other malignancies;

4. Reviews, protocols, short communication, personal opinions, letters,

conference abstracts and laboratory research;

5. Studies whose full texts were not available;

6. Studies published in other languages than English, Portuguese, and Spanish.

7. Duplicate samples.
