# Supplementary material for: Clinicopathological Analysis and Survival Outcomes of Radiation‐Induced Oral Squamous Cell Carcinoma: A Systematic Review and Meta‐Analysis
Source: J Oral Pathol Med. 2025 Dec 30;55(4):448–57. doi: 10.1111/jop.70106 (PMC13065901; doi:10.1111/jop.70106)
Supplement: Supplementary file 7 — Table S3: Clinical‐pathological characteristics of the sample with R‐OSCC of each included study in the SR. [file JOP-55-448-s001.docx]

Supplementary table 3. Clinical-pathological characteristics of the sample with R-OSCC of each included study in the SR.

| Authors Year Country | Sample with R-SOCC | Sex | | Anatomic site of R-SOCC (%) | Clinical Stage (%) | Lymph node status (%) | Margin status (%) | Microscopic information (%) | Treatment (%) |
| --- | --- | --- | --- | --- | --- | --- | --- | --- | --- |
|  |  | Male (%) | Female (%) |  |  |  |  |  |  |
| Chow et al.  2024  China | 60 | 43 (71.7) | 17 (28.3) | Tongue 51 (85.0)  Buccal mucosa 4 (6.7)  Alveolus 1 (1.7)  Floor of mouth 1 (1.7)  Palate 3 (5.0) | I / II 42 (70.0)  III / IV 18 (30.0) | N0 50 (83.3)  N+ 10 (16.7) | Negative 47 (92.2)  Positive 4 (7.8) | **Tumor microscopic differentiation**  Well 15 (26.8)  Moderate 31(55.4)  Poor 10 (17.8)  **Lymphovascular invasion**  Positive 13 (76.5)  Negative 4 (23.5)  **Perineural invasion**  Positive 27 (51.9)  Negative 25 (48.1) | Surgery 51 (85.0)  RT or CT 2 (3.3)  CT 2 (3.3)  Supportive care 5 (8.3) |
| Dai et al.  2020  China | 83 | 67 (80.7) | 16 (19.3) | Tongue 54 (65.1)  Floor of mouth 6 (7.2)  Buccal mucosa 13 (15.7)  Gingiva 10 (12.0) | I / II 51 (61.4)  III / IV 32 (38.6) | N0 55 (66.3)  N+ 28 (33.7) | Negative 83 (100.0)  Positive 0 (0.0) | **Tumor microscopic differentiation**  Well 47 (56.6)  Moderate 23 (27.7)  Poor 13 (15.7)  **Perineural invasion**  Positive 23 (27.7)  Negative 60 (72.3)  Unknown 0 (0.0)  **Lymphovascular invasion**  Positive 16 (19.3)  Negative 67 (80.7)  Unknown 0 (0.0) | Surgery 15 (18.1)  Surgery and RT 30 (36.1)  Surgery, RT and CT 38 (45.8) |
| Hu et al.  2018  China | 116 | 92 (79.3) | 24 (28.9) | Tongue 70 (60.3)  Buccal mucosa 5 (4.3)  Gingiva 27 (23.3)  Palate 14 (12.1) | I / II 69 (59.5)  III / IV 47 (40.5) | N0 98 (84.5)  N+ 18 (15.5) | NDA | **Tumor microscopic differentiation**  Well 64 (55.2)  Moderate 38 (32.7)  Poor 14 (12.1) | Curative 89 (76.7)  RT 6 (5.2)  CT 21 (18.1) |
| Song et al.  2021  China | 48 | 32 (66.7) | 16 (33.3) | Tongue 18 (37.5)  Gingiva 10 (20.8)  Buccal Mucosa 12 (25.0)  Floor of mouth 4 (8.3)  Palate 3 (6.3)  Mandibular bone 1 (2.1) | I / II 13 (27.1)  III / IV 35 (72.9) | N0 36 (75.0)  N+ 12 (25.0) | Negative 39 (81.3)  Positive 9 (18.7) | **Tumor microscopic differentiation**  Well/ moderate 43 (89.6)  Poor 5 (10.4) | Surgery 37 (77.1)  Surgery and RT 1 (2.1)  Surgery and CT 10 (20.8) |
| Toda et al.  2009  Japan | 3 | 2 (66.7) | 1 (33.3) | Gingiva 2 (66.7)  Tongue 1 (33.3) | I / II 3 (100.0) | NDA | NDA | NDA | Brachytherapy 1 (33.3)  Surgery and RT 1 (33.3)  Surgery 1 (33.3) |

Legend:

NDA - No data available

RT - Radiotherapy

CT - Chemotherapy
