# Supplementary material for: Clinicopathological Analysis and Survival Outcomes of Radiation‐Induced Oral Squamous Cell Carcinoma: A Systematic Review and Meta‐Analysis
Source: J Oral Pathol Med. 2025 Dec 30;55(4):448–57. doi: 10.1111/jop.70106 (PMC13065901; doi:10.1111/jop.70106)
Supplement: Supplementary file 8 — Table S4: Summary of clinical‐pathological characteristics of the sample with s‐OSCC. [file JOP-55-448-s007.docx]

Supplementary table 4. Summary of clinical-pathological characteristics of the sample with s-OSCC.

| **Sample Characteristic (n= 1,387)** | **N** | **%** |
| --- | --- | --- |
| **Sex (n=1,387)** |  |  |
| Male | 1063 | 76.6 |
| Female | 324 | 23.4 |
| **Alcohol status (n=1,274)** |  |  |
| Alcoholic drinker | 209 | 16.4 |
| Non-alcoholic drinker | 1065 | 83.6 |
| **Tobacco status (n=1,384)** |  |  |
| Smoker | 413 | 29.8 |
| Non-smoker | 971 | 70.2 |
| **Anatomic site (n=1,387)** |  |  |
| Tongue | 659 | 47.5 |
| Gingiva | 177 | 12.8 |
| Buccal mucosa | 213 | 15.3 |
| Floor of mouth | 313 | 22.6 |
| Palate | 24 | 1.7 |
| Alveolus | 1 | 0.1 |
| **Clinical Stage (AJCC) (n=1,387)** |  |  |
| Stage I-II | 759 | 54.7 |
| Stage III-IV | 628 | 45.3 |
| **Lymph node status (n=1,387)** |  |  |
| N0 | 942 | 67.9 |
| N+ | 445 | 32.1 |
| **Margin status (n=70)** |  |  |
| Positive | 7 | 10.0 |
| Negative | 63 | 90.0 |
| **Tumor microscopic differentiation (n=1,322)** |  |  |
| Well | 637 | 48.2 |
| Moderate | 489 | 37.0 |
| Poor | 196 | 14.8 |
| **Perineural invasion status (n=1,181)** |  |  |
| Positive | 156 | 13.2 |
| Negative | 1025 | 86.8 |
| **Lymphovascular invasion status (n=1,212)** |  |  |
| Positive | 166 | 13.7 |
| Negative | 1046 | 86.3 |
| **Treatment (n=1,355)** |  |  |
| Surgery alone | 215 | 15.9 |
| Surgery combined with radiotherapy and/or chemotherapy | 981 | 72.4 |
| Radiotherapy alone | 5 | 0.4 |
| Chemotherapy alone | 17 | 1.2 |
| Radiotherapy or Chemotherapy | 34 | 2.5 |
| Curative treatment* | 95 | 7.0 |
| Supportive care ** | 8 | 0.6 |

Legend:

AJCC - American Joint Committee on Cancer

* Surgery or surgery plus concurrent chemoradiotherapy.

** The authors did not provide a clear definition of supportive care.
