# Supplementary material for: Clinicopathological Analysis and Survival Outcomes of Radiation‐Induced Oral Squamous Cell Carcinoma: A Systematic Review and Meta‐Analysis
Source: J Oral Pathol Med. 2025 Dec 30;55(4):448–57. doi: 10.1111/jop.70106 (PMC13065901; doi:10.1111/jop.70106)
Supplement: Supplementary file 9 — Table S5: jop70106‐sup‐0009‐TableS5.docx.—Risk of bias assessed using the Joanna Briggs Institute tool for use in Systematic Reviews. The risk of bias was categorized as high, when the study score up to 49% “yes”, moderate when the study scored 50% to 69% “yes”, and low when the study scored more than 70% “yes”. [file JOP-55-448-s002.docx]

**Supplementary Table 5 –** Risk of bias assessed using the Joanna Briggs Institute tool for use in Systematic Reviews. The risk of bias was categorized as high, when the study score up to 49% “yes”, moderate when the study scored 50% to 69% “yes”, and low when the study scored more than 70% “yes”.

**Cohort Studies**

| **Authors** | **Q.1** | **Q.2** | **Q.3** | **Q.4** | **Q.5** | **Q.6** | **Q.7** | **Q.8** | **Q.9** | **Q.10** | **Q.11** | **% yes/risk** |
| --- | --- | --- | --- | --- | --- | --- | --- | --- | --- | --- | --- | --- |
| Chow et al. 2023 | Y | Y | Y | Y | Y | Y | Y | Y | U | U | Y | 81.8%/L |
| Dai et al. 2020 | Y | Y | Y | Y | Y | Y | Y | Y | U | U | Y | 81.8%/L |
| Hu et al. 2018 | Y | Y | Y | Y | Y | Y | Y | N | U | U | Y | 72.7%/ L |
| Toda et al. 2009 | N | N | N | N | N | Y | Y | N | Y | Y | Y | 45.4%/H |
| Song et al. 2021 | N | N | Y | Y | Y | Y | Y | Y | N | N | Y | 72.7%/L |
| **% Yes** | 60% | 60% | 80% | 80% | 80% | 100% | 100% | 60% | 20% | 20% | 100% |  |

Q.1. Were the two groups similar and recruited from the same population? Q.2.Were the exposures measured similarly to assign people to both exposed and unexposed groups? Q.3.Was the exposure measured in a valid and reliable way? Q.4.Were confounding factors identified? Q.5. Were strategies to deal with confounding factors stated? Q.6. Were the groups/participants free of the outcome at the start of the study (or at the moment of exposure)? Q.7. Were the outcomes measured in a valid and reliable way? Q.8.Was the follow up time reported and sufficient to be long enough for outcomes to occur? Q.9. Was follow up complete, and if not, were the reasons to loss to follow up described and explored? Q.10. Were strategies to address incomplete follow up utilized? Q.11.Was appropriate statistical analysis used?

Y-Yes; N- No; U- unclear; H- High, M- Moderate; L- Low.
